# Investigation of Parvovirus B19, Cytomegalovirus, Herpes Simplex Virus Types 1 and 2, Human Papillomavirus Types 16 and 18 in Papillary Thyroid Carcinoma

**DOI:** 10.30699/IJP.2023.1982745.3032

**Published:** 2023-07-16

**Authors:** Fakhriyeh Kalavari, Parin Tanzifi, Tahereh Yousefi, Maryam Lotfi, Elham Nazar

**Affiliations:** 1 *Department of Pathology, Guilan University of Medical Sciences, Razi Hospital, Rasht, Iran*; 2 *Department of Pathology, Douglass Hanly Moir Pathology, New South Wales, Australia*; 3 *Department of Pathology, Amir-Alam Hospital Complex, Tehran University of Medical Sciences, Tehran, Iran*; 4 *Department of Pathology, Sina Hospital, Tehran University of Medical Sciences, Tehran, Iran*

**Keywords:** Cytomegalovirus, Herpes simplex virus, Human papillomavirus, Papillary thyroid carcinoma, Parvovirus B19

## Abstract

**Background & Objective:**

Viral infections are associated with the pathogenesis and progression of human malignancies. Several studies have suggested the role of viral infections in papillary thyroid carcinoma (PTC). However, the results are still conflicting, and the potential role of viruses in PTC tumorigenesis remains to be elucidated. In the present study, we aimed to investigate the presence of parvovirus B19, cytomegalovirus (CMV), herpes simplex virus types 1 and 2 (HSV-1/HSV-2), and human papillomavirus (HPV) types 16 and 18 in PTC.

**Methods:**

In this cross-sectional study, paraffin-embedded tissue blocks of 40 patients with PTC were used. Tissue blocks were studied for the presence of the virus genome using real-time polymerase chain reaction (PCR).

**Results:**

Of the 40 patients with PTC, there was 1 positive case of HPV (2.5%), while 6 cases were positive for parvovirus B19. HSV and CMV DNAs were not detected in any cases.

**Conclusion:**

Correlations among HSV, CMV, and PTC are unexpected in our patient population. But parvovirus B19 and, to a lesser extent, HPV DNA genomes were detected in PTC using real-time PCR.

## Introduction

Papillary thyroid carcinoma (PTC) is the most common type of endocrine malignancy worldwide. It accounts for 90% of all cases of endocrine cancers and 1% of all types of cancer with increasing incidence. This increase may be due to changes in lifestyle or environmental factors and improvements in diagnostic modalities. The disease is attributed to numerous internal and external factors (1). Carcinogenesis of PTC has been associated with many factors, including genetic and familial predisposition, hormonal status of the patient, and body mass index. Many studies have suggested that external factors may play major roles in the pathogenesis, progression, and recurrence of PTC. Environmental factors (such as iodine deficiency or exposure to radioactive radiation) are among the most known etiological factors (2). Recently, attention has turned to the study of oncogenic viruses as potential pathogens in PTC tumorigenesis (3). According to the International Agency for Research on Cancer (IARC), about 20% of all cancers are associated with biological carcinogens (4). Previous studies have reported a higher presence of simian vacuolating virus 40** (**SV40) and Epstein-Barr virus (EBV) in PTC specimens compared with normal controls (5). Zhong *et al.* demonstrated a higher incidence of central lymph node metastasis (CLNM) in positive hepatitis B surface antigen (HBsAg) PTC patients than those in negative ones (6). Recent studies have indicated that the parvovirus B19 capsid protein has a higher prevalence in PTC patients compared with normal individuals (3). Moreover, human papillomavirus (HPV) is reported to have a higher prevalence in the PTC group than in the control group (7). Accordingly, it seems there must be a potential link between these viruses and PTC pathogenesis. Since there are a limited number of studies investigating the correlation between the above-mentioned viruses and the occurrence or development of PTC, the need for further assessment and evaluation cannot be denied. The present research was designed to study the presence of parvovirus B19, herpes simplex virus types 1 and 2 (HSV-1/HSV-2), cytomegalovirus (CMV), and HPV types 16 and 18 using real-time polymerase chain reaction (PCR) in PTC patients.

## Material and Methods

**Study Design and Samples**

This cross-sectional study was conducted on patients diagnosed with PTC through tissue assessment from thyroidectomy and sonography reports confirming evidence of malignancy. All eligible patients were recruited from Amir-Alam Hospital, which is affiliated with Tehran University of Medical Sciences, between 2018 and 2020. Exclusion criteria were patients who received Iodine therapy before surgery. Tumor samples were histopathologically categorized based on the World Health Organization (WHO) criteria.

**DNA Extraction, PCR, and Sequencing**

DNA extraction was performed using a Roche extraction kit (Roche, Germany) according to the manufacturer's protocol. Tissue samples were deparaffinized by soaking in xylene for 30 min. Dehydration and subsequent rehydration with absolute ethanol and distilled water were respectively performed. To lyse other proteins, the samples were resuspended in tissue lysis buffer and proteinase K solution. The samples were incubated at 37°C overnight and then incubated for the second time at 55°C for 1-2 hr. After that, the binding buffer and isopropanol were added to the remaining dissolved tissue samples. Then, the insoluble tissue sections were separated, and the liquid sections were transferred to filter tubes and collection tubes. They were centrifuged to extract DNA. AmpliSens kits (AmpliSens, Russia) were used to detect parvovirus B19, HSV-1, HSV-2, CMV, and HPV types 16 and 18 using real-time PCR.

**Statistical Analysis**

The quantitative variables were represented in the form of mean ± SD, while the percentage was used to present qualitative categorical variables. The quantitative variables were compared against one another using a 1-way analysis of variance (ANOVA). If the results had abnormal distribution, the Kruskal-Wallis test was used for comparison. The chi-square test or Fisher’s exact test was used to make comparisons between qualitative variables. Pearson correlation coefficient and Spearman rank correlation tests were used to assess the correlation between quantitative variables. A multivariate logistic regression analysis was used to determine the difference in the presence of the aforementioned viruses in patients. The results were presented in an odds ratio format (95% CI). Statistical analysis was performed using SPSS version 21 (SPSS Inc., Chicago, IL., USA). *P* values less than 0.05 were considered statistically significant.

**Ethical Considerations**

This research was conducted in accordance with the principles of the Helsinki Declaration. All procedures involving human-derived tissue in this study were carried out in compliance with national and international laws and regulations. Informed consent was obtained from the patients.

The Ethics Committee of Tehran University of Medical Sciences (Amir-Alam Hospital) approved this study.

## Results and Discussion

A total of 40 PTC cases were evaluated, with a mean age of 41.66±15.34 years (range, 14 to 75 years). Nine patients were male (21.2%), and 31 (78.8%) were female. Approximately 70% of the participants (28 cases) had unifocal patterns, and 30% (12 cases) had multifocal patterns. The involvement of lymph nodes was reported in 32.5% (13 cases) of all patients. There was 1 positive case of HPV (2.5%), while 6 positive cases of parvovirus B19 (15%) were reported. No positive cases of HSV and CMV were reported in patients. The characteristics of all patients and the results of real-time PCR are shown in Table 1.

Considering the positive cases of HPV in terms of gender, the frequency of positive cases in men and women was 0% and 3.2%, respectively. The frequency of parvovirus B19 infection in men and women was 11.8% and 15.9%, respectively, without any significant difference between the 2 genders (*P*=0.674).

The average age of the patients with positive and negative cases of parvovirus B19 was 38.67 ± 12.92 years and 42.19±15.75, respectively, without any difference between the 2 groups (*P*=0.467). 

The frequency of HPV infection in those with and without lymph node involvement was 0% and 3.7%, respectively. The frequency of parvovirus B19 infection in those with and without lymph node involvement was 15.4% and 14.8%, respectively, without any difference between the 2 groups (*P*= 0.947). 

In terms of the focal pattern, the frequency of HPV infection in those with unifocal and multifocal patterns was 3.6% and 0%, respectively. The frequency of parvovirus B19 infection in those with unifocal and multifocal patterns was 14.3% and 16.7%, respectively, without any difference between the 2 patterns (*P*=0.745). 

Considering the concurrency of viral involvement with HPV and parvovirus B19, no cases of concurrent involvement of the 2 infections were observed in patients. 

The pathogenesis of PTC is not yet fully clarified. Recently, researchers have focused on the implication of oncoviruses in the carcinogenesis of solid tumors, which may mainly be due to improvements in detection techniques. The investigation of tissue involvement in thyroid cancer by all types of the virus has become possible through genetic assessment and determination of the genotypic pattern of viruses’ genomes. This has led to evidence indicating correlations between some viral infections and PTC (8). The role of some infections (such as herpesviruses, parvovirus B19, and CMV) in the occurrence or development of thyroid cancer has been suggested in some studies (9).

**Table 1:** Characteristics and PCR results of the PTC specimens

Patient Number	Age	Sex	Focality	Lymph node Involvement	HPV	Parvovirus B19	HSV	CMV
1	14	F	Unifocal	Negative	_	_	_	_
2	40	F	Unifocal	Negative	_	_	_	_
3	29	M	Multifocal	Positive	_	_	_	_
4	44	F	Unifocal	Negative	_	_	_	_
5	65	F	Unifocal	Positive	_	_	_	_
6	30	F	Unifocal	Positive	_	_	_	_
7	33	F	Unifocal	Negative	_	_	_	_
8	42	F	Multifocal	Negative	_	_	_	_
9	62	F	Multifocal	Negative	_	_	_	_
10	26	F	Multifocal	Negative	_	_	_	_
11	70	M	Unifocal	Positive	_	_	_	_
12	31	M	Unifocal	Positive	_	_	_	_
13	30	F	Unifocal	Negative	_	_	_	_
14	26	F	Unifocal	Positive	_	_	_	_
15	28	M	Unifocal	Positive	_	_	_	_
16	30	F	Multifocal	Positive	_	_	_	_
17	55	F	Multifocal	Positive	_	_	_	_
18	33	F	Unifocal	Negative	_	_	_	_
19	46	F	Unifocal	Negative	_	_	_	_
20	34	F	Multifocal	Negative	_	_	_	_
21	30	F	Unifocal	Negative	_	_	_	_
22	66	F	Multifocal	Negative	_	_	_	_
23	27	F	Unifocal	Negative	_	_	_	_
24	75	F	Unifocal	Negative	_	_	_	_
25	57	F	Multifocal	Positive	_	_	_	_
26	47	F	Unifocal	Negative	_	_	_	_
27	38	F	Unifocal	Negative	_	_	_	_
28	36	F	Unifocal	Negative	_	_	_	_
29	70	M	Multifocal	Negative	_	_	_	_
30	43	M	Unifocal	Positive	_	_	_	_
31	41	F	Unifocal	Negative	_	_	_	_
32	39	M	Unifocal	Negative	_	_	_	_
33	32	F	Unifocal	Negative	_	_	_	_
34	34	F	Multifocal	Positive	_	+	_	_
35	58	F	Unifocal	Positive	_	+	_	_
36	40	F	Unifocal	Negative	_	+	_	_
37	22	M	Unifocal	Negative	_	+	_	_
38	68	F	Unifocal	Negative	+	_	_	_
39	50	F	Unifocal	Negative	_	+	_	_
40	28	F	Multifocal	Negative	_	+	_	_

*Note.* HSV = herpes simplex virus; HPV = human papillomavirus; CMV = cytomegalovirus.

Herpesviruses are enveloped viruses with double-stranded DNA genomes (10). Eight types of herpesviruses have been classified into the alpha, beta, and gamma families. The alpha family consists of herpesvirus types 1 and 2 and varicella zoster virus. CMV and Roseola viruses 6 and 7 are members of the beta family. EBV and Kaposi's sarcoma-associated herpesvirus (KSHV)/human herpesvirus 8 (HHV-8) are members of the gamma family (11). Herpesviruses cause a large range of infections, and almost every person will get infected with these viruses throughout his/her life. The correlation between herpesvirus and thyroid cancer has recently been studied by tracking the virus gene. The majority of the studies focused on HSV-1 and HSV-2 have assessed viruses’ genes and nectin cell adhesion molecule 1 (NECTIN1) as mediators of virus entrance to the thyroid tissue and thyroid tumoral cells. The sensitivity of the thyroid cancer cells to HSV and their correlation with the molecular mechanism of the tumors have been studied by *in vitro* methods (12). A study on 65 malignant and 44 benign thyroid specimens showed that HSV DNA was detected in 39.4% of all the specimens. Investigating benign lesions showed a frequency of 25% for HSV-1, while HSV-2 DNA was observed in just 2% of these lesions. However, HSV DNA was mostly observed in malignant lesions (in 47.7% of malignant tissues). In patients with lymph nodes metastasis, the trail of HSV-2 DNA has been recognized. In cases of follicular thyroid carcinoma (FTC), HSV-1 DNA has mostly been detected. Oncogenic mutations have been reported in 70% of the HSV-positive tumors and 27.2% of the HSV-negative tumors. Immunoreactivity has been observed in 84% of all the HSV-positive tumors, which was limited to the epithelial cells (13).

Parvovirus B19 has a single-stranded DNA consisting of 5596 base pairs (bp) and is a non-enveloped virus. The virus genome encodes 3 types of protein, including non-structural protein 1 (NS1), viral protein 1 (VP1), and VP2, which are capsid proteins (14). NS1 belongs to cytotoxic host cells, while VP1 and VP2 are immunogenic proteins. NS1 has been shown to have a major role in the stimulation of interleukin 6 (IL-6) productions. On the other hand, parvovirus B19 expression in colon, lymphoid, thyroid, synovial, and skin cells are associated with particular alterations in inflammatory genes and the subsequent impact on the cellular microenvironment through inflammatory factors, such as nuclear factor-κB (NF-κB), tumor necrosis factor α (TNF-α), and IL-6. This correlation has been shown *in vivo* and *in vitro* (15). Infection with parvovirus B19 is associated with many viral diseases. Several studies have shown a correlation between B19 infection and some cancer types, such as leukemia (16). However, only a limited number of the studies have been conducted on the correlation between parvovirus B19 and solid tumors, and very few of them have specifically examined the association between parvovirus B19 and PTC. The presence of parvovirus B19 VP1/VP2 and B19 genomes in the papillary tumoral cells of the thyroid has been identified in some studies, which may indicate a possible role of parvovirus B19 on the tumorigenesis of PTC (3,4).

CMV is one of the *Herpesviridae* family members. Patients infected with this virus have various clinical manifestations ranging from those without symptoms to severe involvement, even during the neonatal period (17). Meningoencephalitis, rhinitis, pneumonitis, myocarditis, hepatitis, and enterocolitis are particularly observed among those with a defective immune system (18). Following initial infection, the disease will remain in the hidden phase. The seroprevalence of the infection increases as people grow older. This prevalence reaches 60% in those older than 50 (19). The term is known as “micro-infection,” describing the low levels of CMV infection in patients with cancer. Although the role of CMV in carcinogenesis is still unclear, some of the CMV proteins have some characteristics that contribute to tumor advancement or cellular mutations. The involvement mechanism seems to be correlated with the mitogen-activated protein kinase (MAPK) signal path. There are plenty of sensitive mechanisms used to determine the presence of the CMV genome or its associated antigens (20). In another study, approximately 40% of all cases of the thyroid cancer autopsies have shown symptoms of CMV; as a result, CMV is suspected to play a role in causing or developing thyroid tumors (21). 

In our study, the greatest prevalence in tissues was observed in parvovirus B19 (15%). HPV DNA has also been detected in the PTC specimens, though the prevalence was much less (2.5%). However, no positive case of HSV or CMV was identified in patients. According to these results, only parvovirus B19 and, to a lesser degree, HPV may involve in the pathogenesis of PTC.

There are few studies on the correlation between the occurrence of the above-mentioned viral infections and the possibility and risk of PTC. As our studies and assessments show, no comprehensive study has been conducted on the correlation between some of these viruses, such as HPV. However, in a study conducted by Stamatiou *et al*., which examined the viral sequences of BKV virus, EBV, and HPV in 30 thyroid nodal samples that underwent surgery, no HPV DNA was detected in any of the normal thyroid or thyroid lymph node samples (20). On the other hand, according to a recent study conducted on 159 samples (including 77 tissue samples of benign thyroid nodules and 82 of PTC), which were evaluated by PCR for HPV DNA, positive results were far greater in PTC tissues than in benign nodules (7). In a study conducted by Tsai *et al.*, of the 16 thyroid tumoral samples, 4 were positive in terms of CMV, but the DNA of EBV, HSV-1, HSV-2, HHV-8, and HPV were not observed in these samples (22). These results are in line with our research in terms of the absence of HPV, HSV-1, and HSV-2 but differ in terms of the presence of CMV. Huang *et al.* studied tumoral and non-tumoral tissue samples consisting of 5 cases of follicular adenoma and 40 cases of PTC; no protein and DNA of CMV were observed in any of the tissue samples, which is in line with our research (23). The lack of positive CMV infection cases in our research may be due to the small sample size. Regarding the correlation between parvovirus B19 infections in PTC, Adamson *et al.* reported a higher frequency of parvovirus B19 capsid protein in the PTC patients (88%) (3), compared to our research (15%). In another research by Wang *et al.*, parvovirus B19 DNA was frequently observed in human thyroid tissue, but the frequency of parvovirus B19 was found to be higher in patients with papillary carcinomas (PTC) compared to the control group (4). In a recent study conducted by Etemadi *et al.*, parvovirus B19 DNA was detected in 31 out of 36 cases (86.1%) and 3 out of 12 controls (25%) using the PCR technique, which further supported the association of parvovirus B19 and thyroid cancer (24). Ghasemi *et al.*, in another recent study, found parvovirus B19 DNA in 12 out of 93 patients (12.9%) with thyroid tumors, of whom 79 had PTC, 3 had FTC, 2 had Hurthle cell carcinoma, 1 had anaplastic thyroid carcinoma, and 8 had medullary thyroid carcinoma. However, there was no statistically significant difference between the patients and the control group (25).

Considering the possible correlation between HSV infection and PTC, we failed to find any positive cases in patients. The sample size in our study was limited. Future studies with larger sample sizes are required for further confirmation of our results. Additionally, future studies with different techniques in other ethnicities are required to obtain more convincing evidence on whether these viruses have any role in the pathogenesis of PTC or not.

## Conclusion

In 4 studied viruses (including HPV, CMV, HSV, and parvovirus B19), the DNA genome of parvovirus B19 and, to a lesser degree, HPV was detected in the PTC using real-time PCR. HSV and CMV DNA genomes were negative in the samples.

## Funding

None.

## Conflict of Interest

The authors declare no conflict of interest.

## Consent:

Written informed consent was obtained from the patient for the publication of this case report. A copy of the written consent is available for review by the Editor-in-Chief of this journal on request.
